# Standigm ASK™: knowledge graph and artificial intelligence platform applied to target discovery in idiopathic pulmonary fibrosis

**DOI:** 10.1093/bib/bbae035

**Published:** 2024-02-12

**Authors:** Seokjin Han, Ji Eun Lee, Seolhee Kang, Minyoung So, Hee Jin, Jang Ho Lee, Sunghyeob Baek, Hyungjin Jun, Tae Yong Kim, Yun-Sil Lee

**Affiliations:** Standigm Inc., Nonhyeon-ro 85-gil, 06234, Seoul, Republic of Korea; College of Pharmacy, Ewha Womans University, Ewhayeodae-gil, 03760, Seoul, Republic of Korea; Standigm Inc., Nonhyeon-ro 85-gil, 06234, Seoul, Republic of Korea; Standigm Inc., Nonhyeon-ro 85-gil, 06234, Seoul, Republic of Korea; College of Pharmacy, Ewha Womans University, Ewhayeodae-gil, 03760, Seoul, Republic of Korea; Standigm Inc., Nonhyeon-ro 85-gil, 06234, Seoul, Republic of Korea; Standigm Inc., Nonhyeon-ro 85-gil, 06234, Seoul, Republic of Korea; Standigm Inc., Nonhyeon-ro 85-gil, 06234, Seoul, Republic of Korea; Standigm Inc., Nonhyeon-ro 85-gil, 06234, Seoul, Republic of Korea; College of Pharmacy, Ewha Womans University, Ewhayeodae-gil, 03760, Seoul, Republic of Korea

**Keywords:** target identification, knowledge graph, neural network, idiopathic pulmonary fibrosis, epithelial-mesenchymal transition

## Abstract

Standigm ASK™ revolutionizes healthcare by addressing the critical challenge of identifying pivotal target genes in disease mechanisms—a fundamental aspect of drug development success. Standigm ASK™ integrates a unique combination of a heterogeneous knowledge graph (KG) database and an attention-based neural network model, providing interpretable subgraph evidence. Empowering users through an interactive interface, Standigm ASK™ facilitates the exploration of predicted results. Applying Standigm ASK™ to idiopathic pulmonary fibrosis (IPF), a complex lung disease, we focused on genes (AMFR, MDFIC and NR5A2) identified through KG evidence. *In vitro* experiments demonstrated their relevance, as TGFβ treatment induced gene expression changes associated with epithelial–mesenchymal transition characteristics. Gene knockdown reversed these changes, identifying AMFR, MDFIC and NR5A2 as potential therapeutic targets for IPF. In summary, Standigm ASK™ emerges as an innovative KG and artificial intelligence platform driving insights in drug target discovery, exemplified by the identification and validation of therapeutic targets for IPF.

## INTRODUCTION

The process of developing a new drug, from identifying a target to securing approval from the Food and Drug Administration (FDA), spans 10–15 years and exceeds the cost of $2.6 billion [1]. Nine out of 10 drug candidates entering clinical studies face potential failure across Phase I, II or III clinical trials and the drug approval process [2]. Most clinical trial failures occur in the late stages of development, resulting in substantial financial and societal costs. The primary causes of high failure rates are a lack of efficacy or unmanageable toxicity issues, with stoppages often attributed to inaccurate drug target identification [2]. Efficacy failures were responsible for terminating 48% of Phase II and 55% of Phase III clinical trials [3, 4], underscoring the urgent need for enhanced approaches to selecting more effective drug targets at the early development stage. While it is widely acknowledged that identifying high-potential drug targets with strong clinical efficacy is a critical step in contemporary drug discovery [5], finding actionable therapeutic targets remains a challenging task for several reasons. These challenges include biological complexity, a lack of comprehensive knowledge about the molecular mechanisms underlying various diseases and the heterogeneity of diseases. Moreover, traditional experimental-based drug identification is a time-consuming and expensive method that relies on individual laboratory experiments and available physical resources. To address this issue, there is a need for enhanced approaches to selecting more effective drug targets during the early development stage.

The development of computer-based methods, including artificial intelligence (AI) and machine learning (ML), has been extensively studied to support drug development by narrowing down the scope of experimental targets, shortening the drug discovery and development cycle and reducing experimental costs [6–8]. Recent milestones, such as AI-designed anticancer compounds reaching phase 2/3 clinical trials, underscore AI’s growing impact in the field [9]. Nevertheless, integrating diverse data sources presents technological challenges, necessitating specialized computational frameworks for successful drug development.

One of the promising approaches for AI/ML-driven drug discovery is using a heterogeneous graph consisting of a large amount of various relational information, often referred to as a knowledge graph (KG). Since a variety of early drug discovery tasks (e.g. target identification, drug repurposing) can be translated into a link prediction problem over KG, KG-based methodologies are heavily studied based on recommender system, knowledge graph embedding (KGE) and graph neural networks (GNNs) [10–14]. These studies, however, are simply importing and utilizing successful models from other research domains; therefore, several improvements could be made, such as exploiting the properties of biological KGs more or developing models that give more promising results for biology researchers.

We constructed Standigm ASK™, an AI-assisted platform tailored for identifying novel target genes associated with diseases of interest, based on interpretable subgraph evidence to provide better interpretability and more accurate results to researchers. Using the subgraph evidence and interactive user interface, users can judge the prediction results by estimating the underlying mechanism of action with their biological information, which other KG-based AI models rarely express.

To demonstrate the real-world impact of Standigm ASK™, we applied it to identify novel target candidates for idiopathic pulmonary fibrosis (IPF) (available at https://ipf.standigm.com). IPF is a chronic and irreversible interstitial lung disease of unknown cause. The literature indicates that the median survival time following an IPF diagnosis is short [15, 16]. Additionally, IPF is often diagnosed at a late stage, limiting treatment options and resulting in a poor prognosis [17]. Currently, pirfenidone and nintedanib are the only drugs approved by the FDA for treating IPF. While these drugs can delay disease progression and alleviate symptoms, they don’t offer a cure for IPF or significantly improve survival rates [18, 19]. Moreover, these drugs are associated with side effects such as thrombocytopenia, gastrointestinal discomfort and dermatological reactions [20, 21]. Given these limitations, there is a critical need to identify new therapeutic targets to treat IPF effectively. Although the pathogenesis of IPF remains unclear, existing studies have reported that EMT plays a crucial role in its development and progression.

In this study, given the crucial role of EMT in IPF development and progression, we identified a ranked set of IPF drug targets (AMFR; Autocrine Motility Factor Receptor, MDFIC; MyoD Family Inhibitor Domain Containing and NR5A2; Nuclear Receptor subfamily 5 group A member 2) through Standigm ASK™ and complementary DNA (cDNA) microarray analysis. As an experimental validation, the results showed that treatment with TGFβ, an EMT activator and fibrosis inducer, increased both mRNA and protein levels of these genes in L132 cells. Small interfering RNA (siRNA) knockdown of these genes activated E-cadherin promoter activity and inhibited mesenchymal cell marker expression, reversing TGFβ-induced EMT-related changes. Moreover, gene knockdown inhibited TGFβ-induced morphological changes and cell migration without affecting cellular proliferation. These findings identify *AMFR*, *MDFIC* and *NR5A2* as potential novel therapeutic targets for IPF treatment through regulating the EMT pathway.

Taken together, Standigm ASK™ has fundamentally reshaped the drug discovery and development paradigm. In this context, we elucidate the process involved in identifying novel targets for IPF throughout this platform’s conceptualization and implementation stages, along with the validation results. This study underscores the significant impact of Standigm ASK™ in expediting the drug discovery process.

## MATERIAL AND METHODS

### KG construction

KGs are often constructed for drug discovery tasks, including target identification, because KGs can have type information in their nodes and edges with the different components referred to as metanode and metaedge [14] that give the interconnectivity of biomedical systems to drug discovery prediction models. We built a KG connecting multimodal biomedical data retrieved from public repositories [22–61] (see Table 1 for public data sources).

**Table 1:** List of public databases used by Standigm ASK™ KG

Name	Data types
Bgee	expressed_low, expressed_high
BindingDB	binds_to
BioGRID	PPI
ChEMBL	binds_to
ClinGen	associated
CTD	binds_to, downregulated_by, treats, upregulated_by, associated
DISEASES	associated
DisGeNET	associated
DrugCentral	PC, binds_to, treats, categorized_in
EFO	DI
ERC	covaries
FAERS	SE, causes
GO	GO, biological_process, cellular_component, molecular_function
GWAS Catalog	associated
Harmonizome	downregulated_in, upregulated_in
IntAct	PPI
LINCS Connectivity Map	downregulated_by, upregulated_by, associated, KD_downregulates, KD_upregulates, OX_downregulates, OX_upregulates
MEDLINE	occurs_in, presents, mentioned_with
NCBI Genes	GE
MINT	PPI
Open Targets	associated
Pathway Commons	PPI
PDSP Ki Database	binds_to
PharmacotherapyDB	treats
Reactome	PW, involved_in, PPI
STARGEO	downregulated_in, upregulated_in
STRING	PPI
TRRUST	PDI
Uberon	AN
*Misc.*	PPI

### Label construction for target prioritization

**Figure 1:**
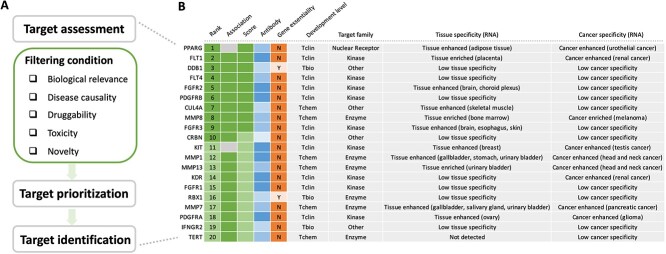
Our workflow of target identification. (**A**) Targets are assessed based on five strategic criteria. The five criteria are individually quantified, and a global score is then computed to prioritize the top targets for which the overall rationale is the highest. Target identification is further performed independently for each disease based on several flexible filters. (**B**) Top 20 hits for IPF. The ranking of the targets and additional filters are applied to refine the list to satisfy the objectives of the user’s study. The high color intensity in the heatmap stands for high ratings.

To assess the large amount of information in the KG of Standigm ASK™, some metrics have been established to rationalize the assessment and prioritization of actionable therapeutic targets. We selected five strategic summary criteria: biological relevance, disease causality, druggability, toxicity and novelty (Figure 1A).

The five criteria are individually quantified and assigned a global score to identify potential disease-associated therapeutic targets. For example, a list of 20 genes with the highest ranking was extracted for IPF. Drug targets were first prioritized based on the given information, including disease association (rank and association score), druggability (Protein Data Bank, small molecules and antibodies), development filters (active max phase and development level), target family, tissue specificity and toxicity filters (gene essentiality and cancer specificity). Then, the number of potential target candidates is narrowed down to highly promising targets associated with the disease. Finally, individual ‘target candidates’ are generated to represent the overall assessment visually and quickly interpretably to end users for any given target (Figure 1B).

### Pre-training representation of KG

Standigm ASK™ uses QuatE [62] as the underlying KGE model, which is known for its ability to resolve complex relationships, including symmetry, anti­symmetry and inversion, by using the score functions defined on the quaternion domain (see Supplementary Material available online at http://bib.oxfordjournals.org/ for detail). Note that we used KGE as preprocessed data for Standigm ASK™ to improve performance on the target discovery task and to provide supportive information selected from the KG instead of making predictions based on the results of KGE alone.

### Metapath selection and path extraction

In graph theory, a path is defined as a sequence of edges where neighboring edges must be connected through a common node [63]. In the KG of Standigm ASK™, we defined a path using the same definition and additionally defined a type of path (called metapath) as the metaedge sequence of the edges in the path. Standigm ASK™ extracts paths between a given query node and a retrieved node and then uses those paths to learn whether an actual edge exists.

However, using all possible paths can lead to an exponential increase in required computing resources and a decrease in explanatory power, so only a few crucial paths were extracted and used. Briefly, we established the following procedure. First, select a set of metapaths by solving a data-driven optimization problem, where the optimization problem is designed to remove redundant or irrelevant metapaths. Then, for each chosen metapath, we pick top-k paths based on the path score (see Supplementary Material available online at http://bib.oxfordjournals.org/ for details).

### Neural network architecture

**Figure 2:**
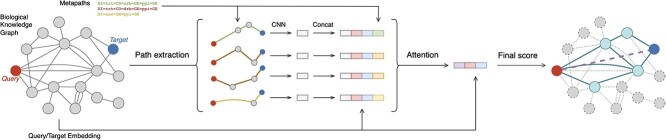
Details of Standigm ASK™ model. Given a query disease and a target gene, Standigm ASK™ extracts a set of paths between the query and the target based on predefined metapaths. Each path is passed through a convolutional neural network and transformed into a path vector. The path vectors are concatenated with the query vector, target vector and metapath vector and then merged into a single-feature vector based on attention mechanism. Finally, the feature vector, query and target vector are used for the final score prediction.

By applying the above methods, we can extract the information needed for further investigation for each query-retrieval pair. However, it still needs to be more practical to manually search the evidence paths between a given query and thousands of candidates. Therefore, we designed a neural network trained to predict the existence of an association (Figure 2). We can perform the first filtering round by excluding candidates with low prediction scores.

The underlying neural network requires three inputs: information for the query, information for the retrieval and the extracted paths between the query and the retrieval. For the query and the retrieval information, Standigm ASK™ directly uses the node embedding vectors obtained from the pre-trained KGE model. A path is processed to a path vector, computed from the embedding vectors of the nodes belonging to it. Specifically, the node vectors are stacked, passed through a convolutional neural network and then converted into a single path vector. Path vectors are then aggregated into a single feature vector using an attention mechanism to obtain different weights of importance for each path depending on the context. Finally, we can compute the association score using the query, target and feature vectors (see Supplementary Material available online at http://bib.oxfordjournals.org/ for details).

### Training with ranking loss

In a typical classification problem, a model is trained using cross­entropy loss by conceptualizing the model’s score as a probability. However, in the link prediction task on the graph, it is sometimes hard to distinguish whether the lack of association is ‘truly negative’ or ‘positive but not yet observed’. This implies that the negative data in the training set might be false negative, which could be potentially problematic. Furthermore, some association edges in our KG contain information on causality, and the causal edges over the association edges were prioritized.

Thus, we adopted a different approach from the recommender system. When a query is provided, Standigm ASK™ attempts to learn the rank of the target by increasing the difference in scores for the following cases: (i) a target that is known to have a causal relationship with a given query and the other is just associated with it (casual versus association) and (ii) a target known to have an association with a given query and the other does not (association versus no association). Loss functions designed for this kind of problem include Bayesian personalized ranking loss (BPR; [64]) and weighted approximately ranked pairwise loss (WARP; [65]). We used WARP loss for training (see Supplementary Material available online at http://bib.oxfordjournals.org/ for details).

### Performance comparison

The following simple comparative analysis was performed to demonstrate the superior performance of our proposed model. We first applied QuatE on our KG and measured the predictive performance for the baseline. Then, we trained Standigm ASK™ and other well-known state-of-the-art models using the pre-trained embeddings. For the state-of-the-art models, we chose neural factorization machine (NFM) [66], relational graph convolutional network (R-GCN) [67] and graph attention network (GAT) [68] with modification from Kamiński *et al*. [69] to handle edge features (here, we'll denote it as EGAT). Notice that we do not use external features (e.g. genetic sequences of genes, textual explanation of diseases, …) for every method. All models share the same fixed QuatE node embedding and were trained with the same optimizer, same loss function and same hyperparameters.

The performance metric for every query disease using average precision (AveP) and precision at 20 (Prec@20) was evaluated with two different ground truth test sets: (i) using all association edges as the ground truth and (ii) using causal edges only. The training set and test set were split using the following procedure. For each query disease, 50% of DI = asw = GE edges were randomly picked as the test edge. If the number of test edges associated with a specific query disease was less than 20, all such test edges were excluded from the test set. All association edges used for training were removed entirely for every evaluation case, and the training/test set was shared for each experiment.

### Application for IPF target identification

Following the prioritization of the top hypotheses on candidate targets using the Standigm ASK™ scoring system, the rationale is subsequently consolidated through a deep-dive investigation by biologists and pharmacologists. During this consolidation phase, an extensive literature review and in-house translational data analyses are performed to confirm that the identified target candidates are involved in specific disease pathways and are druggable with a specific compound modality.

Researchers can then validate target hypotheses by experimentally confirming that disease activity is impacted following perturbation of the target of interest using various biological/chemical approaches. Wet-lab gene inhibition (e.g. via CRISPR-Cas9 deletion or RNA silencing) or preclinical experiments using cellular assays or animal models are commonly implemented to support the hypothesis that drugs interacting with the target exhibit the anticipated pharmacological activity.

For IPF, the score for every target was computed, and all but the top 10% of targets were removed. Secondary filtering was then performed by giving each subgraph a specific condition. These conditions can vary, for example, ‘contains a certain gene’ or ‘has a certain metapath’.

### Experimental methods for validating IPF targets

#### Cell culture

A human normal lung epithelial cell line (L132) and HEK 293T cells were supplied by the American Type Culture Collection (Rockville, MD, USA) and cultured in RPMI (Gibco, Gaithersburg, MD, USA) or DMEM supplemented with 10% fetal bovine serum and 1% penicillin–streptomycin at 37°C in a humidified 5% CO_2_ incubator.

#### siRNA transfection and TGFβ1 treatment

siRNAs against GP78 (sc-43809), MDFIC (sc-89686), NR5A2 (sc-37897) and a control siRNA (sc-37007) were purchased from Santa Cruz Biotechnology (Dallas, TX, USA). For transient siRNA transfection, L132 cells were plated and incubated for 24 h to reach 70% confluency. The cells were then transfected with the designated siRNAs (60 nM) in each experiment using Lipofectamine 3000 (Invitrogen, Carlsbad, CA, USA) and OPTI-MEM (Gibco), in accordance with the manufacturers’ protocols. Transforming growth factor-beta 1 (TGFβ1) was purchased from Bio-Techne R&D Systems (240-B-002; Minneapolis, MN, USA) and cells were treated with 5 ng/ml TGFβ1.

#### Microarray experiment

In accordance with previously described methods [70], total RNA from mouse lung tissues was prepared using the Easy-Spin™ total RNA extraction kit according to the manufacturer’s instructions (iNtRON Biotechnology, Seoul, Republic of Korea). Before performing the microarray experiment, the quality of the purified RNA was measured using the Agilent 2100 Bioanalyzer (Agilent Technologies, Santa Clara, CA, USA); only samples with an RNA integrity number greater than 7.0 were included in the microarray analysis. RNAs from triplicate experiments at each time point were pooled to exclude experimental bias. Isolated total RNA was amplified and labeled using the Low RNA Input Linear Amplification kit PLUS (Agilent Technologies, Santa Clara, CA, USA) and then hybridized to a microarray containing approximately 44 000 probes (~21 600 unique genes), in accordance with the manufacturer’s instructions (Agilent Mouse Whole Genome 44K, Agilent Technologies). The arrays were scanned using an Agilent DNA Microarray Scanner (Agilent Technologies).

#### RNA isolation and qRT-PCR

Total RNA was isolated from the sample using TRIzol® reagent (Qiazen, Valencia, CA, USA). RNA purity and concentration were measured with a Nanodrop. The RNA was reverse-transcribed using a ReverTra Ace® qPCR RT Kit (Toyobo, Osaka, Japan), in accordance with the manufacturer’s protocol. PCR was performed to assess expression of the candidate genes using primers designed for mRNA sequences. mRNA expression was assessed using real-time PCR with an SYBR Green PCR Master Mix kit (Bioline USA Inc., Taunton, MA, USA) and CFX96 Touch™ Real-Time PCR Detection System (Bio-Rad, Hercules, CA, USA), equipped at Ewha Drug Development Research Core Center. The 2^−ΔΔCt^ method was used to analyze the relative changes in gene expression based on real-time quantitative PCR. *Gapdh* was used as an internal control gene. Reaction conditions started with enzyme activation at 95°C for 2 min, followed by 40 cycles of 95°C for 5 s, 58°C for 10 s and 72°C for 20 s. The primer sequences for qRT-PCR are listed in Supplementary Table 2 available online at http://bib.oxfordjournals.org/.

#### Luciferase reporter assay

HEK293T cells were seeded at a density of 1.5 × 10^5^ cells/dish in 35 mm cell culture dishes. The cells were transiently transfected with 0.3 μl of an E-cadherin promoter plasmid DNA for 3 h, followed by transfection of designated siRNAs. After 24 h of incubation, the cells were treated with TGFβ1 (5 nM) for 24 h and E-cadherin promoter activity was measured using the Luciferase Assay System with Reporter Lysis Buffer (E4030; Promega, Madison, WI, USA).

#### MTT assay

Cell proliferation upon the transfection of siRNAs and treatment with TGFβ was determined using an MTT [3-(4,5-dimethylthazol-2-yl)-2,5-diphenyl tetrazolium bromide] assay (M5655; Sigma-Aldrich, St. Louis, MO, USA) in 96-well plates. L132 cells were seeded at a density of 4 × 10^3^ cells/well and treated with siRNAs for 24 h, followed by TGFβ treatment for 12, 24 and 48 h. Then, at different time points, cells were incubated with MTT (final concentration 5 mg/ml) for 4 h in an incubator. Then, the medium was carefully removed and 100 μl of DMSO was added to each well to solubilize the cells. The absorbance was measured on a microplate reader (Tecan, Männedorf, Switzerland), equipped at Ewha Drug Development Research Core Center. At least three independent experiments were performed.

#### Immunoblotting

For immunoblotting, cells were lysed with RIPA lysis buffer (Biosesang, Incheon, Republic of Korea). Protein concentration was determined by the Bradford method (Bio-Rad). The samples were boiled for 5 min, and an equal amount of protein was analyzed by SDS-PAGE (6–15%) using standard conditions. The horseradish peroxidase (HRP) activity was measured using enhanced chemiluminescence (EzWestLumi, Tokyo, Japan) at Ewha Drug Development Research Core Center. Protein band intensity was visualized on ChemiDoc (Bio-Rad) and quantified using ImageJ software 1.45 (National Institutes of Health, Bethesda, MD, USA).

#### Antibodies and reagents

Protein levels were detected using commercial antibodies as follows: MDFIC, NR5A2, N-Cadherin, β-actin (Santa Cruz Biotechnology); AMFR (Proteintech, Rosemont, IL, USA); E-cadherin (BD Biosciences, Santa Clara, CA, USA); α-SMA (Sigma-Aldrich); snail, slug (Cell Signaling Technology, Danvers, MA, USA); twist (Abcam, Cambridge, UK); and β-catenin, Alexa488-conjugated phalloidin (Invitrogen). The details of the antibodies used for immunoblotting and immunofluorescence staining are provided in Supplementary Table 3 available online at http://bib.oxfordjournals.org/.

#### Phalloidin staining of F-actin

To observe changes of the actin cytoskeleton, fluorescence-conjugated phalloidin staining was performed. After siRNA transfection and TGFβ treatments, cells were fixed and permeabilized. To visualize the actin cytoskeleton, F-actin was stained by Alexa Fluor™ 488 Phalloidin for 90 min in the dark. Then, the nuclei of the stained cells were counterstained with DAPI (F6057; Sigma-Aldrich) and stained cells were imaged using a Zeiss Apotome (Carl Zeiss, Oberkochen, Germany), equipped at Ewha Drug Development Research Core Center.

#### Wound healing assay

For monolayer wound healing assays, transfected cells were plated in 6-well dishes to reach confluency of 90% (for 24 h) and 80% (for 48 h). Treatment with TGFβ (5 nM) was applied for 24 h, and parallel wounds of 1 mm were made using an SPLScar (SPL, Gyeonggi-do, Republic of Korea). The sizes of the wounds after 24 and 48 h were measured using a light microscope (Carl Zeiss) in three independent experiments.

#### Statistical analysis

Data were analyzed using GraphPad Prism 5.0 (GraphPad Software Inc., San Diego, CA, USA). The statistical significance of differences with the control group was determined by Student’s *t*-test. The differences were considered statistically significant at *P* ≤ 0.05, *P* ≤ 0.01 and *P* ≤ 0.001.

## RESULTS

### Construction of Standigm ASK™

**Figure 3:**
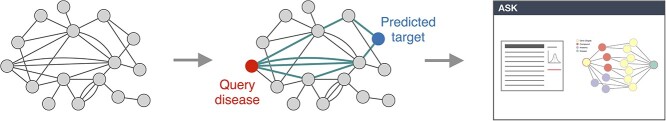
Standigm ASK™ general framework. Data sources are curated and integrated into a KG. For each application starting from one disease or a set of diseases, the KG is mined to evaluate the predicted targets, generate hypotheses and assist the consolidation.

Figure 3 provides a summary of Standigm ASK™, the platform that we used to discover novel targets. It involved three steps: (1) building a KG from the relevant biological data from different sources; (2) applying algorithms to the graph to generate and rank hypotheses about new targets or drug repurposing; and (3) postprocessing with subgraph evidence and exploiting the knowledge of researchers on an interactive user interface.

**Figure 4:**
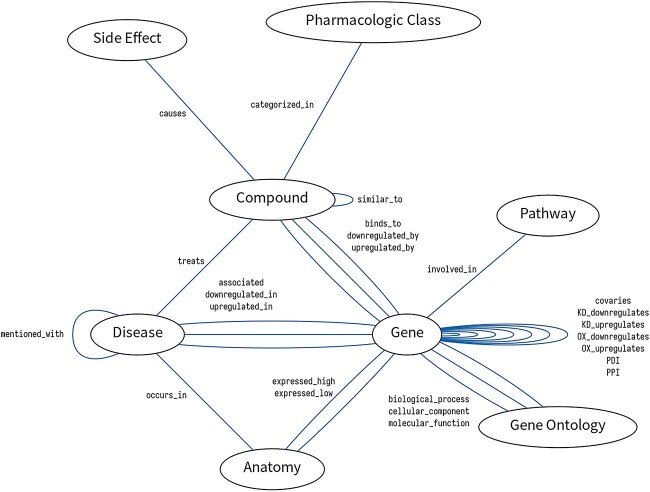
KG schema of Standigm ASK™.

In this study, we employed a KG consisting of 73 227 nodes categorized into 8 metanodes and 4 425 359 edges categorized into 25 metaedges (Figure 4 and Supplementary Table 1 available online at http://bib.oxfordjournals.org/). As shown in Table 2, the contained information on KG used by Standigm ASK™ is comparable to other well-known biological KGs [10, 14, 71–74]. Based on the KG, we obtain a list of metapaths as in Table 3. The KG is further combined with supplementary attributes (e.g. fold changes, *P*-values) and enriched with semantics or ontologies to facilitate navigation through the concepts (e.g. GO, ChEBI, EFO and MedDRA), although we do not employ these attributes in this study.

**Table 2:** Simple quantitative comparison with other biological KGs

KG name	Number of nodes	Number of metanodes	Number of edges	Number of metaedges	Number of sources
Standigm ASK™	73 K	8	4.4 M	25	36
HetioNet	47 K	11	2.2 M	24	29
DRKG	97 K	13	5.7 M	107	34
BioKG	105 K	10	2 M	17	13
PharmKG	7.6 K	3	500 K	29	7
OpenBioLink	184 K	7	4.7 M	30	17
Clinical Knowledge Graph	16 M	35	220 M	57	35

**Table 3:** List of selected metapaths

DI = mnw = DI = asw = GE	DI = mnw = DI = uri = GE
DI = asw = GE > pdi > GE	DI = asw = GE = ppi = GE
DI = oci = AN = oci = DI = asw = GE	DI = trt = CO = trt = DI = asw = GE
DI = trt = CO = trt = DI = uri = GE	DI = trt = CO = bin = GE = ppi = GE
DI = trt = CO = drb = GE = ppi = GE	DI = trt = CO = urb = GE = ppi = GE
DI = asw = GE = bin = CO = drb = GE	DI = asw = GE = bin = CO = urb = GE
DI = asw = GE = drb = CO = drb = GE	DI = asw = GE = drb = CO = urb = GE
DI = asw = GE = inv = PW = inv = GE	DI = dri = GE = ppi = GE
DI = dri = GE = bin = CO = drb = GE	DI = dri = GE = bin = CO = urb = GE
DI = uri = GE = drb = CO = bin = GE	DI = uri = GE = inv = PW = inv = GE

The five strategic criteria, biological relevance, disease causality, druggability, toxicity and novelty, confer biological actionability to Standigm ASK™. Biological relevance is based on cumulative evidence predicting that a gene or protein is relevant due to biomolecular associations or dysregulations. Genes with high biological relevance tend to cluster and form disease modules within the KG. Disease causality assesses whether a target is a cause or consequence in the observed pathophysiology. This can be determined by analyzing genetic associations, expression of the relevant cells or tissues (e.g. from GTeX) and AI/ML predictions. The causality of each data plays an important role when training the model. Druggability is the likelihood of modulating the function of a target with either small synthetic or biological drugs. This is assessed using the measure proposed by Open Targets [45] based on clinical trial data, discovery experiments and computational predictions. In addition, toxicity is related to the potential toxic implications of interfering with a given target. Finally, novelty can be determined through clinical trial data, patents and literature mining using natural language techniques (NLP).

### Performance of Standigm ASK™

**Figure 5:**
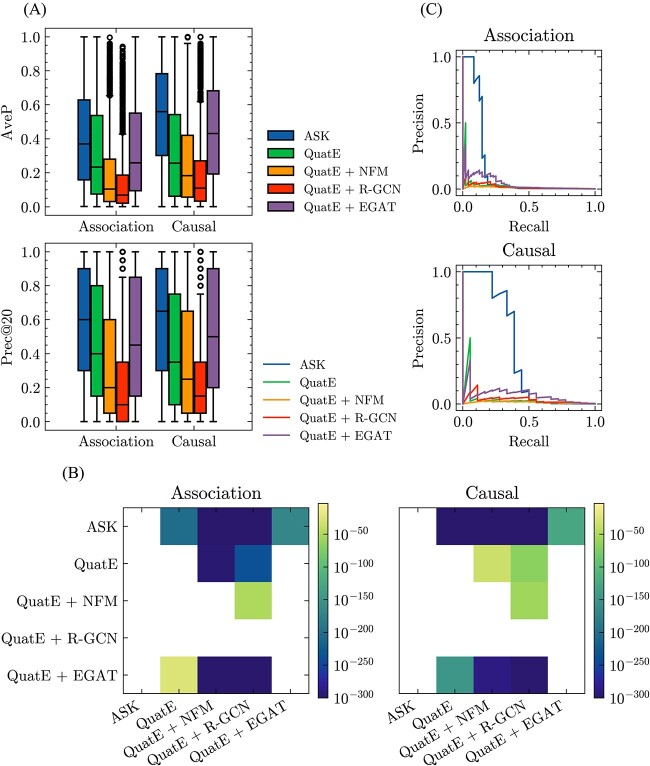
(**A**) Performance comparison of prediction association/causal edges between naive models on KGEs versus Standigm ASK™. Average precision (AveP) and precision at 20 (Prec@20) are evaluated for each disease query and displayed as boxplots. (**B**) *P*-values for one-sided Wilcoxon signed-rank tests on AveP. The *x*-axis represents the baseline method, and the *y*-axis represents the comparison method. Non-significant performance improvement (*P*-value >0.001) is not colored. (**C**) Precision–recall curves calculated from predicted scores of IPF.

Figure 5 indicated that Standigm ASK™ outperformed the other baseline models in terms of both AveP and Prec@20 (Figure 5A), achieving significantly high improvements (Figure 5B). The difference in performance was even more dramatic when limited to IPF, where Standigm ASK™ had an AveP of 0.148 (on all-association ground truth) and 0.378 (on causal-only ground truth). Meanwhile, none of the other baselines had an AveP above 0.1 (Figure 5C).

### Prediction of epithelial–mesenchymal transition-related IPF targets

We selected IPF as a disease of interest for the empirical validation study of Standigm ASK™ because IPF is a progressive and fatal lung disease of unknown cause, and there is still a large unmet clinical need for more efficacious and better-tolerated drugs. Although the exact mechanisms of IPF are unclear, recent studies highlight the significant role of EMT in its development and progression [75, 76]. In the early stages, persistent damage and inflammation prompt epithelial cells to undergo epithelial–mesenchymal transition (EMT), transforming into mesenchymal cells. These mesenchymal cells then differentiate into fibroblasts, fostering fibrosis and interacting with other cells to worsen the fibrotic process. Targeting EMT is, therefore, crucial for the advancement of IPF therapies.

**Figure 6:**
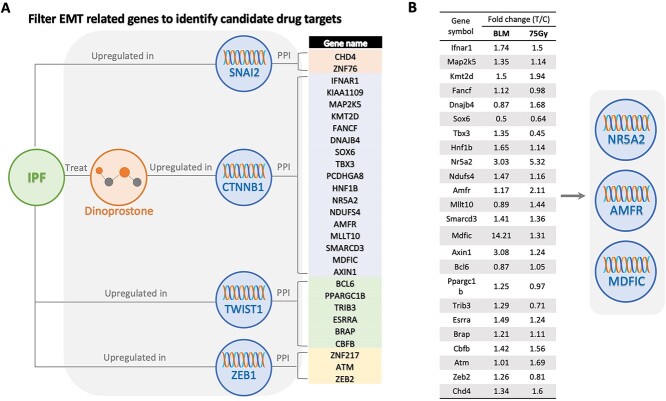
The process of potential IPF target identification. (**A**) Twenty-eight genes were predicted as ‘IPF candidate drug targets’ through filtering with EMT pathway–related gene nodes (SNAI2, CTNNB1, TWIST1 and ZEB1). (**B**) Microarray analysis after treatments of BLM and 75 Gy of radiation in mice showed the upregulated expression of potential targets (NR5A2, AMFR and MDFIC) that can contribute to the development of lung fibrosis.

To establish a set of potential IPF targets related to the EMT pathway, we first obtained a set of genes predicted to be associated with IPF generated through KG learning. Then, exploiting the availability of a learned KG for each gene, we used the characteristics of these gene sets to extract targets, including EMT pathway nodes (*SNAI2*, *CTNNB1*, *TWIST1* and *ZEB1*) from the KGs. Interestingly, four paths were found in all subgraphs: IPF-[urb]-(*SNAI2*)-[ppi]-Targets; IPF-[trt]-(Dinoprostone)-[urb]-(*CTNNB1*)-[ppi]-(Target); IPF-[urb]-(*TWIST1*)-[ppi]-Targets; and IPF-[urb]-(*ZEB1*)-[ppi]-Targets. In these paths, the IPF node is directly connected to either EMT pathway nodes (*SNAI2*, *TWIST1* and *ZEB1*) or dinoprostone (a pulmonary fibrosis regulator that expresses β-catenin), which is linked to *CTNNB1*. In addition, a total of 28 genes were discovered in subgraphs on protein–protein interaction with EMT nodes. As a result, these targets were predicted to be potential drug targets against IPF, acting through regulation of the EMT pathway (Figure 6A).

### Further target filtering with cDNA microarray data

To demonstrate the biological significance of the genes selected through KG learning, we analyzed the cDNA microarray in a pulmonary fibrosis mouse model [70]. Specifically, to identify genes selected for KG learning, we selected genes presenting greater than 2-fold change in expression compared with the control group in mouse models of pulmonary fibrosis induced by 75 Gy radiation or bleomycin (BLM). We selected four genes (*NR5A2*, *AMFR*, *MDFIC* and *AXIN*) that were upregulated more than 2-fold in the pulmonary fibrosis mouse model compared with the levels in the control group. However, we excluded the *AXIN* gene due to its role as both a positive and a negative effector of the Wnt signaling pathway [77, 78]. The genes *NR5A2*, *AMFR* and *MDFIC* were selected from the KG learning and cDNA microarrays, suggesting they may be responsible for IPF (Figure 6B).

### EMT experimental results for new target validation

**Figure 7:**
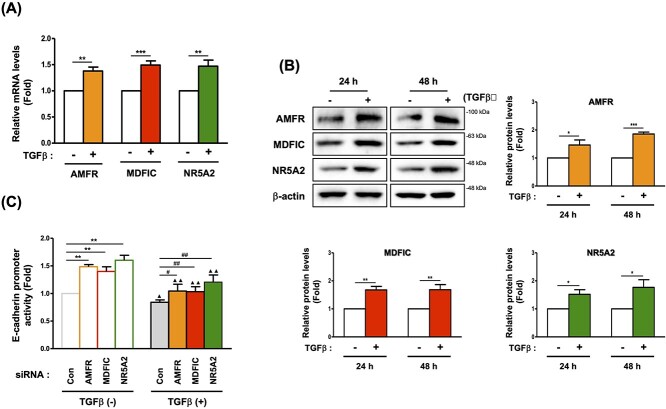
AMFR, MDFIC and NR5A2 could be the targets inducing EMT in lung fibrosis. (**A**) qRT-PCR data of AMFR, MDFIC and NR5A2 after 24 h of treatment with TGFβ. (**B**) Immunoblots of AMFR, MDFIC and NR5A2 after 24 or 48 h of TGFβ treatment. (**C**) E-Cadherin promoter activity after siRNA transfection with or without 24 h of treatment with TGFβ measured by luciferase reporter assay (^*^P < 0.05, ^*^^*^P < 0.01, ^*^^*^^*^P < 0.001 compared with control cells; ^#^*P* < 0.05, ^#^^#^*P* < 0.01, ^#^^#^^#^*P* < 0.001 compared with cells treated with TGFβ alone).

To elucidate whether the *AMFR*, *MDFIC* and *NR5A2* genes are involved in the EMT process, we investigated these genes’ responsiveness to TGFβ, a well-known EMT activator, as well as an inducer of fibrosis [79, 80]. Treatment of L132 lung epithelial cells with 5 nM TGFβ increased these three genes’ mRNA and protein levels, with similar induction rates being identified among the genes (Figure 7A and B). We also examined E-cadherin promoter activity after siRNA transfection of each gene with or without TGFβ treatment. siRNA of *AMFR*, *MDFIC* and *NR5A2* activated E-cadherin promoter activity, with little difference in activation potency among the three genes. Treatment of HEK293T cells with 5 nM TGFβ inhibited E-cadherin promoter activity, but siRNA treatments of the three genes restored this promoter activity (Figure 7C). Furthermore, the western blotting data from the siRNA treatment of all three genes confirmed the regulation of β-catenin expression (Supplementary Figure 1A available online at http://bib.oxfordjournals.org/).

**Figure 8:**
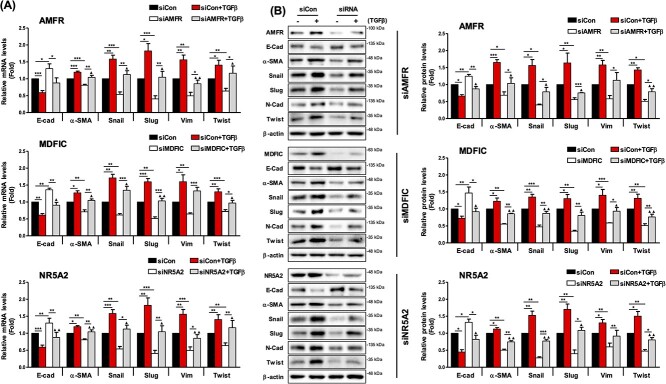
Expression of downstream components associated with lung fibrosis. (**A**) qRT-PCR and (**B**) immunoblot analysis of the mRNA levels of genes associated with lung fibrosis after transfection of designated siRNAs with or without treatment of TGFβ (^*^P < 0.05, ^*^^*^P < 0.01, ^*^^*^^*^P < 0.001; ▲*P* < 0.05, ▲▲*P* < 0.01, ▲▲▲*P* < 0.001; ▲: control (+TGFβ) versus siRNA (+TGFβ).

EMT-related genes such as E-cadherin (epithelial cell marker), β-catenin, α-SMA, Snail, Slug, Twist and Vimentin (mesenchymal cell markers) were also examined using L132 cells. The results showed that TGFβ treatment increased mesenchymal cell markers and inhibited epithelial cell markers; however, siRNA treatment of these genes reversed these trends, with similar effects being identified at the mRNA and protein levels (Figure 8A and B and Supplementary Figure 1B and C available online at http://bib.oxfordjournals.org/). Similar effects were observed when another cell line, such as the human normal bronchial epithelial cell line (BEAS-2B), was used (Supplementary Figure 2 available online at http://bib.oxfordjournals.org/).

**Figure 9:**
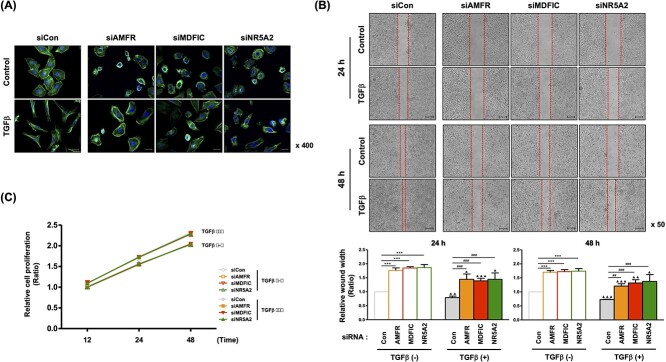
Phenotypes of designated siRNA-transfected cells. (**A**) Effects of siRNAs on F-actin organization in L132 cells in the presence of TGFβ for 24 h. F-actin was visualized by Alexa Fluor™ 488 Phalloidin. The magnification is 40′. (**B**) The migration capacity of siRNA-transfected cells with or without treatment with TGFβ was detected using a wound-healing assay. (**C**) Cell proliferation upon transfection of siRNAs with or without TGFβ treatment was determined by MTT assay (^*^P < 0.05, ^*^^*^P < 0.01, ^*^^*^^*^P < 0.001 compared with control cells; ^#^*P* < 0.05, ^#^^#^*P* < 0.01, ^#^^#^^#^*P* < 0.001 compared with cells treated with TGFβ alone).

Additional experiments were then performed to identify EMT-related morphological changes. Control L132 cells were round or polygonal and exhibited very close cell–cell proximity, reminiscent of cellular tight junctions. Meanwhile, TGFβ treatment transformed the cells into a spindle shape. However, in cells treated with siRNA of the three genes, these TGFβ-induced morphological features were inhibited, with the morphology being restored with similar efficiency among all siRNA treatments (Figure 9A). Wound healing assay also showed that TGFβ-mediated cell migration was dramatically inhibited by the siRNA of each of the three genes, with similar efficiency among all three knocked-down genes (Figure 9B). Regardless of TGFβ treatment, the cellular proliferation rate was not changed by the knockdown of any of the three genes (Figure 9C).

## DISCUSSION AND CONCLUSION

In this study, we introduced Standigm ASK™, a novel AI-assisted drug discovery platform consisting of a KG and a neural network, and we applied it to discover potential targets for IPF and selected three novel genes, *AMFR*, *MDFIC* and *NR5A2*, based on the KG evidence and cDNA microarray analysis. The target prediction for IPF was validated successfully by empirical experiments in an IPF disease cell model in which the inhibition of these predicted target genes by siRNA showed efficacy in wound healing assays. The results revealed that these genes were upregulated by TGFβ, a critical factor in fibrosis and EMT. We also showed that silencing of these genes reversed the EMT-related changes induced by TGFβ, such as changes in cell morphology, cell migration and marker expression, without affecting cell proliferation. These findings indicate that *AMFR*, *MDFIC* and *NR5A2* could be novel therapeutic targets for treating IPF by inhibiting EMT. *MDFIC* enhances cancer stem cell chemoresistance and is implicated in cancer development [81, 82]. Meanwhile, *NR5A2* promotes cancer stem cell properties and tumorigenesis, affecting the prognosis of certain cancers. It has also been reported that BRD4-induced *NR5A2* activation drives the progression of pancreatic cancer [83–85]. Furthermore, *AMFR* promotes myofibroblast differentiation and pulmonary fibrosis, while playing a role in cancer metastasis and EMT [86, 87]. While all three genes impact cancer growth and metastasis, *AMFR* is specifically linked to pulmonary fibrosis [88, 89]. This study is possibly the first to explore the *AMFR*-EMT relationship in IPF, with the findings corroborated by gene set enrichment (GSE) analysis showing *AMFR*’s correlation with the incidence of IPF in humans (Supplementary Figure 3 available online at http://bib.oxfordjournals.org/).

Comparative analysis showed that Standigm ASK™ provides clear benefits in target discovery and outperforms several well-known state-of-the-art models, including NFM, R-GCN and EGAT. Some possible reasons can be proposed to explain this performance gain. First, Standigm ASK™ employs paths as additional features, which contain more valuable contexts and help predict the score. While the general KGE models do not consider the surrounding information and GNNs aggregate every neighboring information of the nodes, Standigm ASK™ can employ the specific context between two nodes, represented as paths. Standigm ASK™ is also expected to perform better on biological KGs because they tend to be denser than common sense KGs, meaning that there are more paths between two nodes; thus, Standigm ASK™ can capture the richer context. Second, Standigm ASK™ focuses on predicting a specific edge type only, so the underlying task becomes more straightforward than the prediction of all edge types.

Nonetheless, there is still substantial room for improvement, most trivially via updating the backbone network architecture into the transformer [90]. The transformer originates from NLP studies but has recently emerged as the most powerful model, being applied in almost every domain (e.g. ViT [91] on images, AlphaFold [92] on proteins). Note that the transformer takes a sequence as an input, and Standigm ASK™ takes paths as input where the path is a sequence of nodes and metaedges extracted from KG. Since both models employ data sequences, Standigm ASK™ has a very favorable structure for applying the transformer backbone. In addition, recent works have proved that the transformer can effectively learn multimodal domains of data (e.g. CLIP [93] on images and languages), which is also a promising result for KGs. Instead of utilizing heterogeneous features in KGs as described in [10–12], Standigm ASK™ can adopt a multimodal transformer for employing these features. Another potential improvement to the model is refining the metapath selection algorithm. There is no good way of preventing the appearance of nonsense metapaths in the current method. A potential solution is to frame it as a human-in-the-loop problem [94], where the algorithm proposes initial candidates and the domain experts select the proposed metapaths. The process can be repeated until a specified number of metapaths have been selected.

KGs have considerable value in the pharmaceutical industry due to the importance of analyzing and integrating heterogeneous biomedical data [95]. The properties of KGs, such as versatility, visualization and compatibility to ML, have accelerated the creation of numerous KG-based models for diverse drug discovery tasks ranging from drug repurposing to target identification, adverse drug reaction prediction and omics data analysis [95–98]. However, some problems, such as insufficient data quality, potential security risks, complexity of the biomedical ontology and inadequate validation methods, remain and impede the real-world applications of the models by the pharmaceutical industry. Data quality issues and potential security risks have been continuously emphasized in the KG application [95–97, 99, 100]. The multifaceted issues of data quality in data extraction and curation, bias, data poisoning and dataset update have been considered substantially by us and others and mitigated by diverse solutions, including the new NLP technology development [101, 102], domain experts assignment [103], stepwise bias-mitigating framework [104], adversarial training [105] and automatic updating system for primary dataset sources, respectively. The ontology problems, such as acronyms, homonyms and the hierarchy of biomedical terminology, have been improved, but we still encounter problems in KG-based models, requiring a unified multimodal biomedical ontology system for ML [101, 106, 107]. Most of all, validation methods are important to KG-based drug discovery models for real-world applications. We successfully demonstrated the practical performance of Standigm ASK™ with empirical biological data, but the wet lab validation process usually demands significant time and cost to seek the relevant biological system and test assays; therefore, the robust experimental design should be accompanied by KG construction and algorithm design as well as *in silico* validation metrics. Lastly, considering the specificity and complexity of biomedical knowledge in drug discovery research, we address the development of contextualized KGs to realize personal medicine in the near future [108].

Comparing Standigm ASK™ with existing KGs, we emphasize that Standigm ASK™ is well balanced in composition and well equipped with documentation quality. As shown in Table 2, Standigm ASK™ has suitable numbers of nodes and edges despite the biggest number of sources in the table, which facilitates efficient learning. Standigm ASK™ meets the evaluation categories in the recent review of Bonner *et al.* [96]; schema, relation explanation and dataset filtering for documentation quality of Standigm ASK™ were clarified in Figure 4, Table 1 and Supplementary Table 1 available online at http://bib.oxfordjournals.org/, respectively. Moreover, although none of the KGs was updated in the review, we have been updated for internal use and are currently testing an automatic database updating and extension to various other data sources, which includes in-house private data.

The potential uses of Standigm ASK™ vary because of its comprehensive multimodal KG. On-target repurposing based on target identification will be easily performed without additional algorithm changes in the platform [95]. Synthetic lethality can be predicted by learning about the local morphology of two specific gene nodes in KG and the known synthetic lethality pairs under particular cell lines or diseases [109]. Predicting synergetic drug combinations can also be realized because large-scale combination screening data such as DrugCombDB [110] and Oncology-Screen [111] are publicly available. Integrating the screening data to the multimodal KG of Standigm ASK™ will provide us with the necessary relations on top of the existing relations such as drug–protein, drug–disease and protein–protein connections, which facilitates the application of ML algorithms for synergetic drug combination [112, 113].

Overall, Standigm ASK™ is a promising approach for novel target discovery. Here, we successfully applied it to identifying novel targets of IPF, demonstrating the qualification for real-world application. Our KG-based platform will shed light on drug target identification, easing the cumbersome drug discovery process caused by complex and enormous biomedical knowledge. Further research will be needed to confirm the universal power of Standigm ASK™ in the other types of drug discovery tasks, such as drug repurposing, synthetic lethality and drug combination predictions.

**Key Points**

- Standigm ASK™ is an artificial intelligence–aided platform that suggests novel target genes for diseases using a heterogeneous knowledge graph and a neural network model.
- The platform was applied to identify three genes (*AMFR*, *MDFIC* and *NR5A2*) as potential targets for idiopathic pulmonary fibrosis, a lung disease that involves epithelial–mesenchymal transition (EMT).
- The three genes were validated by experiments showing their role in modulating EMT and fibrosis in response to TGFβ, an EMT activator and fibrosis inducer.

## Supplementary Material

suppl_figures_bbae035

suppl_tables_bbae035

suppl_model_bbae035

## Data Availability

The top 3000 targets of IPF computed by Standigm ASK™ and related information, including subgraph evidence, are publicly accessible at https://ipf.standigm.com.

## Author Biographies

**Seokjin Han** was an AI scientist at Standigm Inc. at the time of the work.

**Ji Eun Lee** is a master’s course student at Ewha Womans University. Her research interest is the target elucidation of lung fibrosis.

**Seolhee Kang** is a senior scientist at Standigm Inc.

**Minyoung So** is a principal scientist at Standigm Inc. She is interested in AI-based drug discovery, including drug target identification, drug repurposing and drug combination prediction.

**Hee Jin** is a postdoctoral researcher at Ewha Womans University. Her research interests focus on the identification and elucidation of targets in radiation-induced lung fibrosis.

**Jang Ho Lee** is a senior AI engineer at Standigm Inc.

**Sunghyeob Baek** is an AI engineer at Standigm Inc.

**Hyungjin Jun** is a principal scientist at Standigm Inc.

**Tae Yong Kim** was a principal scientist at Standigm Inc. at the time of the work.

**Yun-Sil Lee** is a full professor in the college of pharmacy at Ewha Womans University. Her research interests are the basic radiation damage response in relationship with cancer development and pulmonary fibrosis.
