# Supplementary material for: Standigm ASK™: knowledge graph and artificial intelligence platform applied to target discovery in idiopathic pulmonary fibrosis
Source: Brief Bioinform. 2024 Feb 12;25(2):bbae035. doi: 10.1093/bib/bbae035 (PMC10862655; doi:10.1093/bib/bbae035)
Supplement: suppl_figures_bbae035 [file suppl_figures_bbae035.docx]

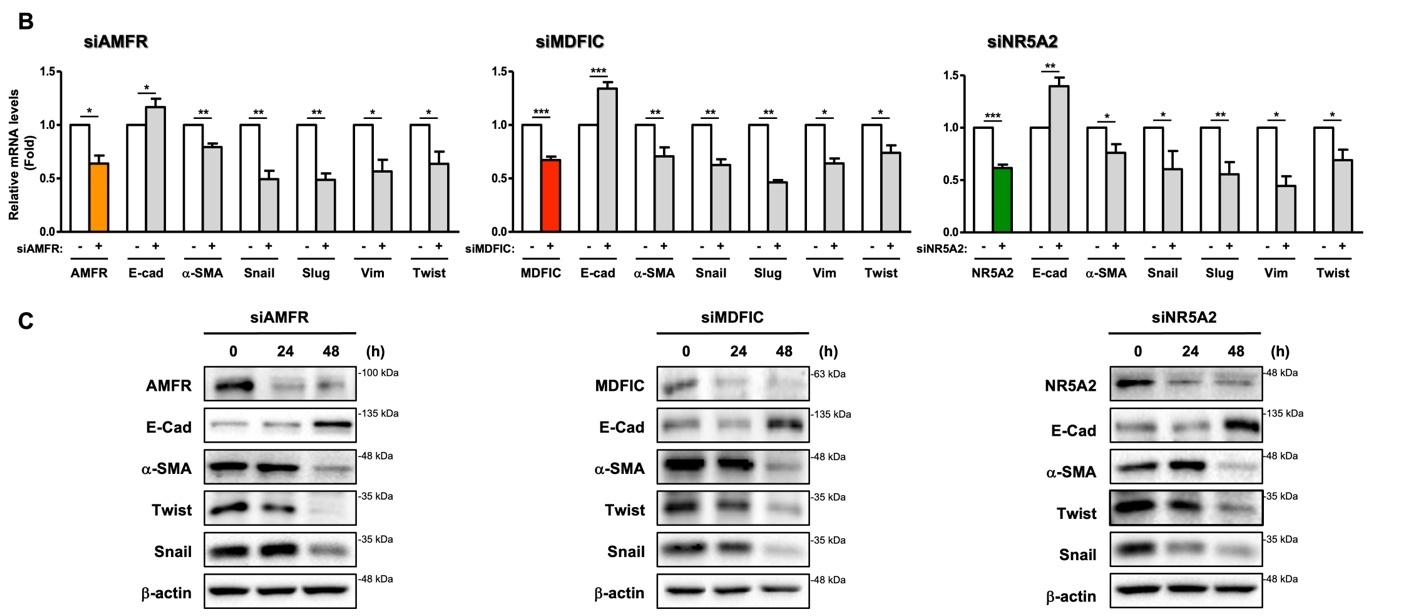

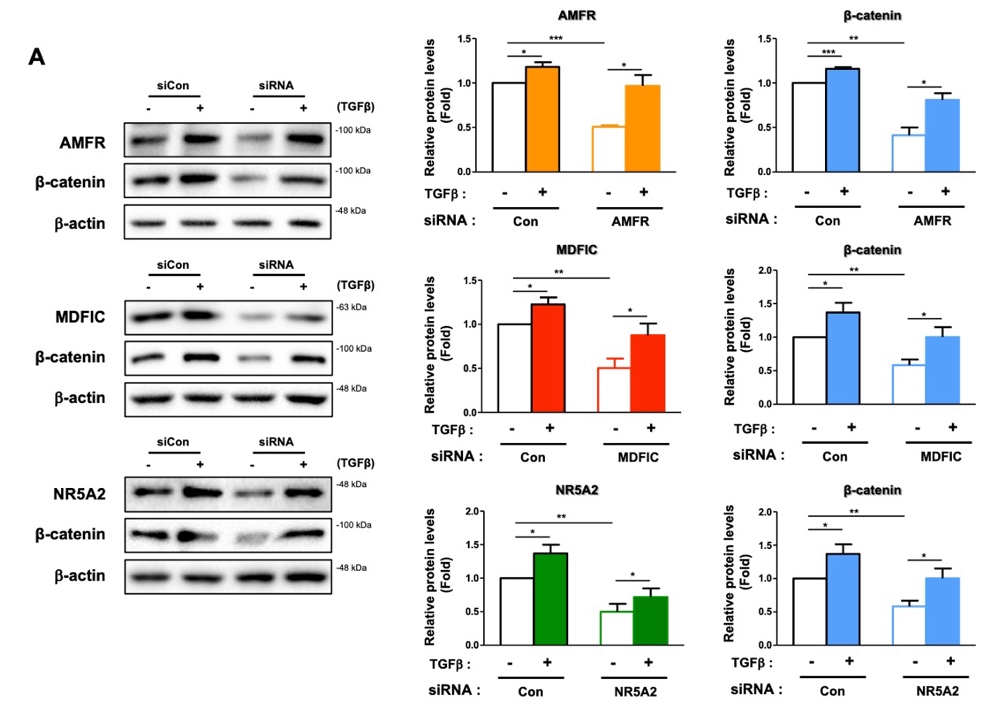


Supplementary Figure 1: Expressions of downstreams associated with lung fibrosis in L132 cells. (A) Immunoblots of β-catenin after transfection of designated siRNAs with or without 24 hours treatment of TGFβ. (B) qRT-PCR (C) immunoblots of downstreams associated with lung fibrosis after transfection of designated siRNAs for 24 hours. (*p< 0.05, **p< 0.01, ***p< 0.001 compared with TGFβ untreated control cells)


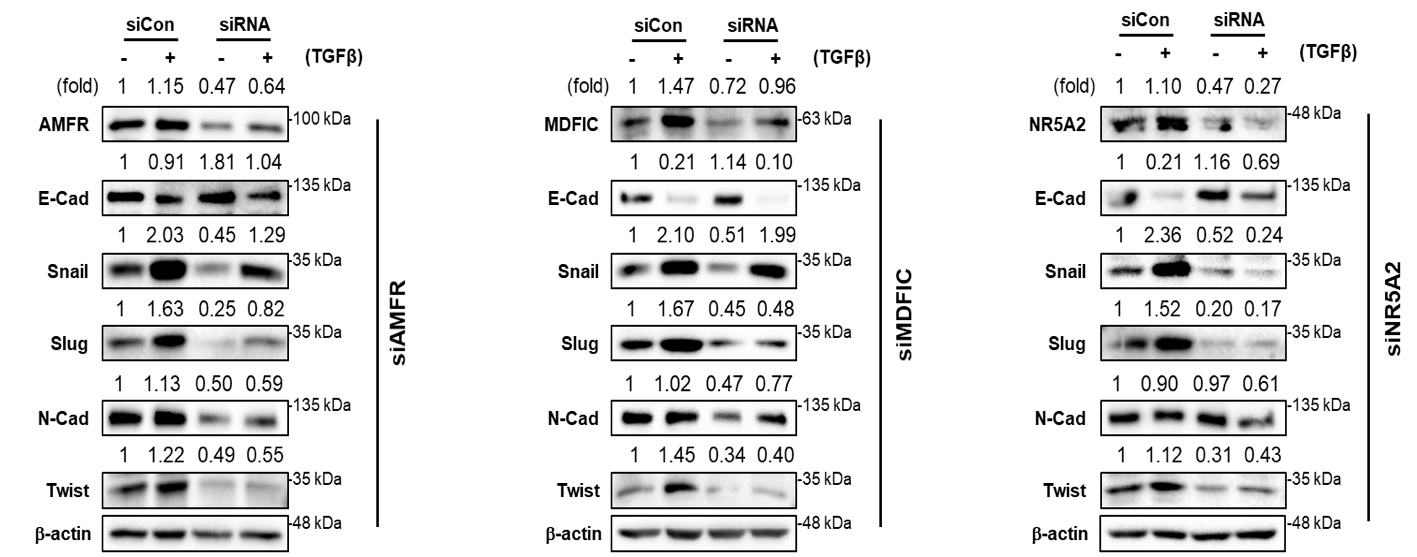


**Supplementary Figure 2: Expressions of downstream components associated with lung fibrosis in Beas-2B cells. Transfection of designated siRNAs with or without treatment of TGFβ using human normal bronchial epithelial cells (Beas-2B). Western blot analysis in Beas-2B cells after transfection of siRNAs with or without TGFβ. The fold increase of each protein blot was normalized to the endogenous control β-actin.**


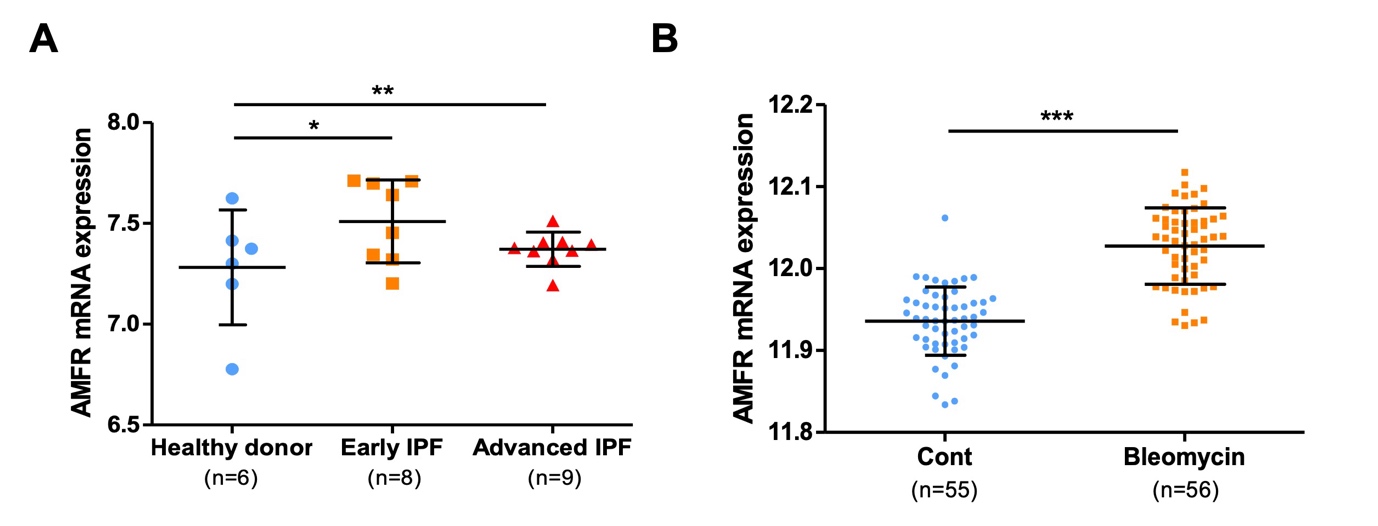


Supplementary Figure 3: GSE (Gene Set Enrichment) analysis. GSE24206 and GSE40151 publicly available gene expression datasets used for therapeutic response rate analysis, were obtained from the National Center for Biotechnology Information (NCBI) gene expression omnibus database (GEO). (A) Analysis of AMFR mRNA expression levels in healthy donor lung tissues (n=6), lung tissues of early IPF patients (n=8), and lung tissues of advanced IPF patients (n=9), analyzed using the GSE24206 dataset. (B) Analysis of AMFR mRNA expression levels in lung tissues of PBS control mice (n=55) and bleomycin-induced lung fibrosis mice (n=56) analyzed using the GSE40151 dataset. (*p< 0.05, **p< 0.01, *** p< 0.001 compared with control group)
