# Supplementary material for: Standigm ASK™: knowledge graph and artificial intelligence platform applied to target discovery in idiopathic pulmonary fibrosis
Source: Brief Bioinform. 2024 Feb 12;25(2):bbae035. doi: 10.1093/bib/bbae035 (PMC10862655; doi:10.1093/bib/bbae035)
Supplement: suppl_tables_bbae035 [file suppl_tables_bbae035.docx]

Supplementary Table 1: Statistics of Standigm ASK™ knowledge graph.

| **Metanode** | **Abbreviation** | **# of nodes** |
| --- | --- | --- |
| Anatomy | AN | 648 |
| Compound | CO | 4,848 |
| Disease | DI | 10,918 |
| Gene | GE | 30,995 |
| Gene Ontology | GO | 14,153 |
| Pharmacologic Class | PC | 1,420 |
| Pathway | PW | 2,344 |
| Side Effect | SE | 7,901 |
| *Total* |  | *73,227* |
|  |  |  |
| **Metaedge** | **Abbreviation** | **# of edges** |
| associated | DI=asw=GE | 772,214 |
| binds_to | CO=bin=GE | 35,565 |
| biological_process | GE=bip=GO | 81,787 |
| categorized_in | CO=cat=PC | 21,207 |
| causes | CO=cau=SE | 57,567 |
| cellular_component | GE=cec=GO | 58,329 |
| covaries | GE=cov=GE | 63,098 |
| downregulated_by | CO=drb=GE | 95,448 |
| downregulated_in | DI=dri=GE | 513,101 |
| involved_in | GE=inv=PW | 105,549 |
| KD_downregulates | GE>kdd>GE | 194,979 |
| KD_upregulates | GE>kdu>GE | 132,251 |
| mentioned_with | DI=mnw=DI | 331,953 |
| molecular_function | GE=mof=GO | 49,793 |
| occurs_in | AN=oci=DI | 105,027 |
| OX_downregulates | GE>oxd>GE | 18,161 |
| OX_upregulates | GE>oxu>GE | 22,346 |
| PDI | GE>pdi>GE | 8,367 |
| PPI | GE=ppi=GE | 950,178 |
| similar_to | CO=sim=CO | 8,656 |
| treats | CO=trt=DI | 56,806 |
| upregulated_by | CO=urb=GE | 94,785 |
| upregulated_in | DI=uri=GE | 489,482 |
| expressed_high | AN=xph=GE | 83,417 |
| expressed_low | AN=xpl=GE | 75,293 |
| *Total* |  | *4,425,359* |

Supplementary Table 2: Primer sequences.

| Gene | Species | Sequence |
| --- | --- | --- |
| AMFR | Human | Forward: AAC TCT GGT GCT CCT GCA TC |
|  |  | Reverse: AAC CCA CAG GCT TTA CGT GC |
| MDFIC | Human | Forward: CAA GAG TTC GAG GCC TTC C |
|  |  | Reverse: ATC ACA TTT TCC CTG GGC TGT |
| NR5A2 | Human | Forward: GCT TTA AGC CAA AGA ACT GCC TA |
|  |  | Reverse: CCC AGC ACC AAT AGG TGT AAG T |
| α-SMA | Human | Forward: GAG AAG AGT TAC GAG TTG CCT GA |
|  |  | Reverse: TGT TAG CAT AGA GGT CCT TCC TG |
| gapdh | Human | Forward: GGG TGT GAA CCA CGA GAA AT |
|  |  | Reverse: ACT GTG GTC ATG AGC CCT TC |
| slug | Human | Forward: CAA CGC CTC CAA AAA GCC AA |
|  |  | Reverse: ACT CAC TCG CCC CAA AGA TG |
| E-Cadherin | Human | Forward: TGC CCA GAA AAT GAA AAA GG |
|  |  | Reverse: GTG TAT GTG GCA ATG CGT TC |
| snail | Human | Forward: CTT CTC ACT GCC ATG GAA TTC CCT |
|  |  | Reverse: GCC TTT GTC CTG TAG CTC AAA GCA |
| twist | Human | Forward: GTC CGC AGT CTT ACG AGG AGC |
|  |  | Reverse: GCT TGA GGG TCT GAA TCT TGC T |
| vimentin | Human | Forward: GAG AAC TTT GCC GTT GAA GC |
|  |  | Reverse: GCT TCC TGT AGG TGG CAA TC |

Supplementary Table 3: Antibody information.

| Antigen | Host | Supplier | Product number |
| --- | --- | --- | --- |
| MDFIC | Mouse | Santa Cruz Biotechnology | sc-515212 |
| NR5A2 | Mouse | Santa Cruz Biotechnology | sc-393369 |
| AMFR | Rabbit | Proteintech | 16675-1-AP |
| β-catenin | Mouse | Invitrogen | ma1-2001 |
| E-Cadherin | Mouse | BD Biosciences | 610181 |
| N-Cadherin | Mouse | Santa Cruz Biotechnology | sc-59987 |
| α-SMA | Mouse | Sigma-Aldrich | A5228 |
| snail | Mouse | Cell Signaling Technology | 3895S |
| slug | Rabbit | Cell Signaling Technology | 9585S |
| twist | Rabbit | Abcam | ab50581 |
| β-actin | Mouse | Santa Cruz Biotechnology | sc-47778 |
| Alexa Fluor 488-phalloidin |  | Invitrogen | A-12379 |
