# Supplementary material for: Standigm ASK™: knowledge graph and artificial intelligence platform applied to target discovery in idiopathic pulmonary fibrosis
Source: Brief Bioinform. 2024 Feb 12;25(2):bbae035. doi: 10.1093/bib/bbae035 (PMC10862655; doi:10.1093/bib/bbae035)
Supplement: suppl_model_bbae035 [file suppl_model_bbae035.docx]

# Supplementary Material

## Notations

Denote $\mathcal{G=}\left( \mathcal{V,E} \right)$ as a knowledge graph consisting of $\left| \mathcal{V} \right|$ nodes and $\left| \mathcal{E} \right|$ relations. We denote $\mathcal{N}$ and $\mathcal{R}$ as the sets of metanodes and metaedges. Then, every edge in $\mathcal{E}$ can be represented as a triplet $\left( h,r,t \right)\mathcal{\in V\times R\times V}$, where $h$, $r$ and $t$ is called head, relation, and tail respectively. Note that we can consider the inverse relations $r^{-1}$. For all $h,r\in\mathcal{V}$, if $\left( h,r,t \right)\mathcal{\in E}$ then $\left( t,r^{-1},h \right)\mathcal{\in E}$. For given edge $e\mathcal{\in E}$, we denote $h\left( e \right)$, $r\left( e \right)$ and $t\left( e \right)$ as the head, relation, and tail of the edge $e$ respectively.

A path $p$ in the knowledge graph is a finite composition of edges; $p=e_{1}\circ e_{2}\circ\ldots\circ e_{l}$ where the tail node of $e_{i}$ and the head node of $e_{i+1}$ is equal for every $i=1, 2,\ldots, l-1$. Similarly, we can define a metapath $P$ as the composition of metaedges: $P=r_{1}\circ r_{2}\circ\ldots r_{l}$ where the tail metanode of $r_{i}$ and the head metanode of $r_{i+1}$ is equal for every $i=1, 2, \ldots, l-1$. If the path $p$ have a metapath $P$, we denote as $p\in P$. For the path $p=e_{1}\circ e_{2}\circ\ldots\circ e_{l}$, we call $l$ is the length of the path $p$. Furthermore, we define the source of the path $p$ as $s\left( p \right)=h\left( e_{1} \right)$ and the destination of the path $p$ as $d\left( p \right)=t\left( e_{l} \right)$.

## Knowledge Graph Embedding

We’ve trained 24-dimension QuatE [1] using GraphVite [2]. Specifically, Adam [3] has been used for optimizer during 1000 epochs, while the number of negatives is set to 64. Other hyperparameters are tuned via NNI [4] (see Table 1).

Table 1: Hyperparameters used for pre-training QuatE model.

| Hyperparameter | Value |
| --- | --- |
| Learning rate | 0.00015969270433620372 |
| Learning rate multiplier for relation | 0.0007585353260151453 |
| Adversarial temperature | 0.21637979548575267 |
| $\mathcal{l}_{3}$ penalty | 0.0010888311359075276 |

## Metapath Selection

Let $\mathcal{P=}\left\{ P_{1},P_{2},\ldots, P_{k} \right\}$ be the candidate set of metapaths. To define redundant or irrelevant metapaths among $\mathcal{P}$, we first define the coverage $C_{P}=\left\{ \left( x,y \right) | \exists p:p\in P, s\left( p \right)=x,d\left( p \right)=y \right\}$ as the set of covered query-retrieval pair $\left( x,y \right)$ of metapath $P$. Similary, define $C_{r}=\left\{ \left( x,y \right):\left( x,r,y \right)\mathcal{\in E} \right\}$ for relation $r$. We consider the metapath $P$ is relevant to the metaedge $r$ if $C_{P}$ and $C_{r}$ are similar. We consider the two metapath $P$ and $P'$ are redundant if $C_{P}$ and $C_{P'}$ are similar.

Let’s think about the intuition behind the above definition. If the coverage of a metapath is exactly same as the coverage of a metaedge, then the presence of that metapath can be used to accurately predict the metaedge. So, if the coverage of a metapath is close to the coverage of the given relation, one can consider the metapath is useful for predicting the relation. In practice, however, it’s extremely unlikely that a single metapath will have the same coverage with a given relation, so we should choose multiple metapaths to use. In this case, we want to remove as many redundant metapaths as possible.

Let $\boldsymbol{s}=\left( s_{i} \right)_{i=1,\ldots,k}\in\mathbb{R}^{k}$ be a similarity vector where $s_{i}$ denotes a similarity between $C_{P_{i}}$ and $C_{r}$, and $\boldsymbol{D}=\left( D_{ij} \right)_{i,j=1,\ldots,k}\in\mathbb{S}^{k}$ be a pairwise distance matrix where $D_{ij}$ denotes a difference between $C_{P_{i}}$ and $C_{P_{j}}$ ($\mathbb{S}^{k}$ is the set of $k\times k$ semidefinite matrices). Recall that $\boldsymbol{s}$ and $\boldsymbol{D}$ are the measures of redundant and irrelevant mentioned above. Currently, we use the variance of information [5] for distance and normalized mutual information [6] for the similarity.

Then, we can design the following optimization problem to maximizes the similarity between the metapath set and the relation, while each selected metapaths are not so similar:

$$\mathrm{maximize} \boldsymbol{s}^{\top}\boldsymbol{x} w.r.t \boldsymbol{x}\in\left\{ 0,1 \right\}^{k}$$

$$sub. to 2\leq\sum_{i=1}^{k} x_{i}\leq m, D_{ij}\geq\gamma\forall i<j:x_{i}=x_{j}=1.$$

where 𝑚 is the maximum size of the metapaths set and 𝛾 is the minimum distance cutoff. The problem is nonconvex and discrete, but we can relax the problem to another convex continuous problem as followed:

$$\mathrm{maximize} \boldsymbol{s}^{\top}\boldsymbol{x} w.r.t. \boldsymbol{x}\in\left[ 0,1 \right]^{k}, \boldsymbol{X}\in\mathbb{S}^{k}$$

$$sub. to \boldsymbol{1}^{\top}\boldsymbol{x}\leq m, \left[ \begin{matrix} 1 & \boldsymbol{x}^{\top} \\ \boldsymbol{x} & \boldsymbol{X} \end{matrix} \right]\in\mathbb{S}_{+}^{k+1}, \left( \boldsymbol{D}-\gamma\boldsymbol{11}^{\top} \right)\circ\left( \boldsymbol{X}-\mathrm{diag}\left( \boldsymbol{x} \right) \right)\geq0,\mathrm{diag}\left( \boldsymbol{X} \right)=\boldsymbol{x}.$$

We solved the optimization problem using CVXPY [7]. After obtaining the relaxed solution $\boldsymbol{x}^{\boldsymbol{*}}$, we sample the actual index vectors from binominal distribution with parameter $\boldsymbol{x}^{\boldsymbol{*}}$.

## Path Extraction

First, compute scores of every edge in the KG using the trained KGE model. Then, for each metapath that was chosen before, paths corresponding to the metapath were extracted using beam search, and pick$k$ paths with the top path score, where the path score is defined as the sum of edge scores belonging to the path. We set $k$ to 2 and beam width to 1000.

## Model Structure

Assume that the model tries to predict the probability of triple $\left( h,r,t \right)$ using selected metapaths $P_{1},P_{2},\ldots, P_{m}$ and extracted paths $p_{1}^{\left( 1 \right)},\ldots,p_{n_{m}}^{\left( m \right)}$ where $p_{i}^{\left( j \right)}$ denotes $i$-th path from the metapath $P_{j}$. Define $X_{e},X_{r}\in\mathbb{R}^{4d}$ be the pretrained QuatE embedding vectors of entity $e$ and relation $r$, and $X_{P}\in\mathbb{R}^{4d}$ be the trainable metapath embedding vector. Note that a quaternion consists of four real numbers, so $d$-dimensional quaternion can be converted into $4d$-dimensional real vector.

Standigm ASK™ first converts each path into a vector form. For each path $p=h\circ e_{1}\circ\ldots\circ e_{l-2}\circ t$, Standigm ASK™ first converts the path into a stacked embedding $\tilde{X_{p}}=\left[ X_{h};X_{e_{1}};\ldots;X_{e_{l-2}};X_{t} \right]\in\mathbb{R}^{4d\times l}$. Then, the stacked embedding passed through the following neural network to obtain $Z_{p}\in\mathbb{R}^{4d}$:

$$Z_{p}=\text{MaxPool}\left( \text{Conv1D}\left( \text{Mish}\left( \text{Conv1D}\left( \tilde{X_{p}} \right) \right) \right) \right).$$

Note that the kernel sizes are set to 2, and the latter convolution layer has no bias term. We use Mish [8] as the activation function.

After computing $Z_{p_{1}^{\left( 1 \right)}},\ldots Z_{p_{n_{m}}^{\left( m \right)}}$, they are aggregated into a single path feature vector using attention mechanism, to weight the importance of each path differently depending on the context. For each $Z_{p}$, the corresponding head embedding, tail embedding, and the metapath embedding are concatenated: $\tilde{Z_{p}}=\left[ Z_{p},X_{h},X_{t},X_{P} \right]\in\mathbb{R}^{16d}$. Note that the head and tail embedding are obtained from the pretrained KGE model and not trainable. Then, the energy $E_{p}\mathbb{\in R}$ and the attention $\alpha_{p}\mathbb{\in R}$ for each path $p$ is computed as followed:

$$E_{p}=W_{2}^{\left( path \right)}\text{Mish}\left( W_{1}^{\left( path \right)}\cdot\tilde{Z_{p}}+b_{1}^{\left( path \right)} \right),$$

$$\alpha_{p}=\frac{\exp\left( E_{p} \right)}{\sum_{p^{'}\in\left\{ p_{1}^{\left( 1 \right)},\ldots,p_{n_{m}}^{\left( m \right)} \right\}} \exp\left( E_{p^{'}} \right)}.$$

Once the attention values are computed, use them to compute the path feature vector $Z^{\left( path \right)}\in\mathbb{R}^{4d}$ as the weighted sum of $Z_{p}$:

$$Z^{\left( path \right)}=\sum_{p^{'}\in\left\{ p_{1}^{\left( 1 \right)},\ldots,p_{n_{m}}^{\left( m \right)} \right\}} \alpha_{p^{'}}Z_{p^{'}}.$$

From the path feature $Z^{(path)}$, we compute the association score $\hat{y}\mathbb{\in R}$ by using co-attention mechanism suggested by [9]. First, we compute head and tail feature vectors $Z^{\left( head \right)},Z^{\left( tail \right)}\in\mathbb{R}^{4d}$ as followed:

$$Z^{\left( head \right)}=X_{h}\odot\text{Sigmoid}\left( W_{1}^{\left( head \right)} \cdot\left[ X_{t},Z^{\left( path \right)} \right]+b_{1}^{\left( head \right)} \right),$$

$$Z^{\left( tail \right)}=X_{t}\odot\text{Sigmoid}\left( W_{1}^{\left( tail \right)}\cdot\left[ X_{h},Z^{\left( path \right)} \right]+b_{1}^{\left( tail \right)} \right).$$

And we can finally compute the association score $\hat{y}$:

$$\hat{y}=W\cdot\left[ Z^{\left( head \right)},Z^{\left( tail \right)},Z^{\left( path \right)} \right]+b$$

In addition to the basic structure described, we applied dropout [10] before every neural networks.

## Loss function

To employ the WARP loss [11], the mini-batch should contain scores from the high priority group $y^{+}\in\mathbb{R}^{b}$ and scores from the low priority group $y^{-,1},\ldots, y^{-,k}\in\mathbb{R}^{b}$. Then, the WARP loss is defined as

$$\mathcal{L}\left( y^{+},y^{-,1},\ldots,y^{-,k} \right)=\frac{1}{b}\sum_{i=1}^{b} \text{ReLU}\left( \max_{j=1,\ldots,k} y_{i}^{-,j}-y_{i}^{+}+\Delta\right)$$

where $\Delta$ is the margin and $\text{ReLU}\left( x \right)=\max\left\{ x,0 \right\}$ is a rectified linear unit function.

## Training

We initialized the model with Kaiming initialization [12] and optimized the model during 50 epochs with Adam [3]. Mini-batch size is fixed to 1024. Furthermore, we add a max-norm constraint while training as described in [10].

## Hyperparameters

Every hyperparameter are listed in Table 2.

Table 2: Hyperparameters used for training.

| Hyperparameters | Value |
| --- | --- |
| Learning rate | 0.001 |
| Dropout probability | 0.1 |
| Max norm penalty | 3.5 |
| Margin (for WARP loss) | 1.0 |
| # of low priority group (for WARP loss) | 3 |

# Reference

1. Zhang S, Tay Y, Yao L, et al. Quaternion Knowledge Graph Embeddings. Advances in Neural Information Processing Systems 2019; 32:

2. Zhu Z, Xu S, Tang J, et al. GraphVite: A High-Performance CPU-GPU Hybrid System for Node Embedding. The World Wide Web Conference 2019; 2494–2504

3. Kingma DP, Ba J. Adam: A Method for Stochastic Optimization. 3rd International Conference on Learning Representations, ICLR 2015, San Diego, CA, USA, May 7-9, 2015, Conference Track Proceedings 2015;

4. Microsoft. Neural network intelligence. 2021;

5. Meilă M. Comparing clusterings—an information based distance. Journal of Multivariate Analysis 2007; 98:873–895

6. Wijaya DR, Sarno R, Zulaika E. Information Quality Ratio as a novel metric for mother wavelet selection. Chemometrics and Intelligent Laboratory Systems 2017; 160:59–71

7. Diamond S, Boyd S. CVXPY: A Python-Embedded Modeling Language for Convex Optimization. J Mach Learn Res 2016; 17:83

8. Misra D. Mish: A Self Regularized Non-Monotonic Activation Function. 2020;

9. Hu B, Shi C, Zhao WX, et al. Leveraging Meta-path based Context for Top- N Recommendation with A Neural Co-Attention Model. Proceedings of the 24th ACM SIGKDD International Conference on Knowledge Discovery & Data Mining 2018; 1531–1540

10. Srivastava N, Hinton G, Krizhevsky A, et al. Dropout: A Simple Way to Prevent Neural Networks from Overfitting. Journal of Machine Learning Research 2014; 15:1929–1958

11. Weston J, Yee H, Weiss RJ. Learning to rank recommendations with the k-order statistic loss. Proceedings of the 7th ACM conference on Recommender systems 2013; 245–248

12. He K, Zhang X, Ren S, et al. Delving Deep into Rectifiers: Surpassing Human-Level Performance on ImageNet Classification. 2015; 1026–1034
